# Effect of pueraria, scutellaria, and coptis decoction for type 2 diabetes: A systematic review and meta-analysis protocol

**DOI:** 10.1097/MD.0000000000019770

**Published:** 2020-04-17

**Authors:** Maoyi Yang, Zhipeng Hu, Rensong Yue

**Affiliations:** Hospital of Chengdu University of Traditional Chinese Medicine.

**Keywords:** and coptis decoction, meta-analysis, protocol, pueraria, scutellaria, systematic review, type 2 diabetes mellitus

## Abstract

**Background:**

Type 2 diabetes mellitus is a common health problem in the world. The overall goal of diabetes management is to control blood glucose and lipids, so as to reduce the incidence of complications. Pueraria, scutellaria, and coptis decoction (PSCD) is 1 of the representative Traditional Chinese medicine formula in the treatment of diabetes, which is widely used in clinical practice in China. At present, there are many clinical reports about this formula, but these reports have their own defects.

Therefore, there is an urgent need for a systematic review and meta-analysis to synthesize the current clinical evidence.

**Methods and analysis:**

A comprehensive literature search will be conducted and randomized controlled trials will be selected according to inclusion and exclusion criteria. Fasting blood glucose and 2 hours postprandial blood glucose will be selected as the main outcomes. The secondary outcomes are glycated hemoglobin, fasting insulin, total cholesterol, glycerol, low-density cholesterol, high-density cholesterol and adverse effects. Chi-square and *I*^2^ test will be used to test the heterogeneity of the study. Subgroup analysis will be conducted to explore the source of heterogeneity and sensitivity will be conducted to test the stability of the results. Funnel plot will be used to evaluate publication bias. Finally, the Grading of Recommendations Assessment, Development and Evaluate system will be used to summarize the quality of evidence.

**Results:**

The results will be published in peer-reviewed journals.

**Conclusion:**

This research will evaluate the efficacy of Pueraria, scutellaria, and coptis decoction in the treatment of type 2 diabetes mellitus patients. It will provide strong evidence-based support for clinical practice.

**OSF registration number:**

DOI 10.17605/OSF.IO/WVDE5

## 1 Introduction

Diabetes is a kind of chronic metabolic disease characterized by continuous high blood glucose. With the development of society and the change of lifestyles, the number of diabetes patients in the world increases year by year.^[1]^ According to the latest 9 International Diabetes Federation diabetes map,^[2]^ 0.463 billion adults aged 20 to 79 all over the world suffers from diabetes. If the current trend continues, there will be 700 million adult diabetics worldwide in 2045. Type 2 diabetes mellitus (T2DM) accounts for more than 90% of diabetic patients. The rapid increase in incidence has led to a marked increase in disease related expenditures. According to International Diabetes Federations estimation, the total amount of global medical expenses related to diabetes have reached 760 billion dollars in 2019. This is 4.5% higher than that in 2017. Among them, America spent 294.6 billion dollars on medical expenses related to diabetes, which was the country with the largest expenditure in the world in 2019. Due to the high morbidity of diabetes and health expenditure, it has brought a very heavy burden to the development of social economy and a significant impact to the health of patients. High prevalence of diabetes and complications seriously affect patients’ physical condition and quality of life. How to prevent and treat diabetes and its complications more effectively has become a hot topic of medical research.

Traditional Chinese medicine has a long history and rich experience in the treatment of T2DM. Pueraria, Scutellaria, and Coptis Decoction (PSCD), founded by Chinese ancient doctor Zhang Zhongjing, is 1 of the representative prescriptions for the treatment of T2DM. It includes four kinds of medicine: Pueraria, Scutellaria, Coptis and Liquorice.^[3]^ The PSCD was found to contain 12 effective ingredients by liquid chromatography-series mass spectrometry, that is puerarin, soybean flavone, baicalin, hanbaicalin, hanbaicalin, gandillin, gandillin, halberine, parmatine, engaging in and glycyrrhizic acid.^[4]^ In animal research, PSCD can regulate intestinal flora, cholesterol related signaling pathway,^[5,6]^ IRS-2 / PI3K-Akt signaling pathway,^[7,8]^ inflammatory cytokines,^[9]^ SIRT1 and other pathways^[10]^ to exert its anti-diabetic effects. In clinical trials, PSCD can reduce blood glucose and lipid, regulate leptin level and stabilize body weight, improve insulin resistance and restore blood glucose stability. Moreover, PSCD combined with other medicines in clinical trials has better efficacy than other medicines alone.^[11]^

However, these studies have some defects, for example, the sample size is small, most of them are single-center studies, and there are biases and unadjusted confounding in these studies. In addition, there is no systematic review or meta-analysis to prove whether PSCD is an ideal alternative therapy. Therefore, this study will conduct meta-analysis to evaluate the clinical efficacy of PSCD on T2DM, expecting to provide high-quality evidence to support clinical practice.

## 2 Methods and analysis

### 2.1 Study registration

We have completed the registration of the systematic review and meta-analysis in the OSF (DOI 10.17605/OSF.IO/WVDE5). This systematic review and meta-analysis protocol is reported according to the Preferred Reporting Items for Systematic Reviews and Meta-analysis Protocols checklist.^[12]^

### 2.2 Inclusion and exclusion criteria

#### 2.2.1 Study design

Our research will only include randomized controled trials. Non- randomized controled trials studies, including animal experiments, retrospective studies and cohort studies will not be included in our research. Likewise, reviews abstract articles and conference articles will not be included.

#### 2.2.2 Participants

Patients with an established diagnose of T2DM will be included in our research. There is no restriction on age, region and gender.

#### 2.2.3 Interventions and comparators

The experimental group takes oral PSCD as the main treatment, without limitation about dosage and form of PSCD. The control group can be blank control group, placebo control group or the control group of conventional treatment for T2DM (such as metformin, insulin, and so on). If the experimental group use combination therapy of PSCD and other medicine or non-medicine therapy (such as acupuncture, massage, etc.), the control group also needs to use the same therapy at the same time.

#### 2.2.4 Outcomes

The primary outcomes are fasting blood glucose and 2-hour postprandial blood glucose. Secondary outcomes will include glycated hemoglobin, fasting insulin, total cholesterol, glycerol, low-density cholesterol, high-density cholesterol and adverse effects.

### 2.3 Study search

We will carry out the study search in eighty databases, including four English Databases: PubMed, Embase, Cochrane Library Central Register of Controlled Trials and Web Of Science, and four Chinese databases: China National Knowledge Infrastructure database, Wanfang Data Knowledge Service Platform, the VIP information resource integration service platform, China Biology Medicine Disc (Sino Med). In addition, we will also search Chinese Clinical Trial Registry (ChiCTR) and ClinicalTrials.gov for ongoing researches. In order to expand the search scope as much as possible, we will also manually search Google scholars, Baidu scholars and the references of the previous published related systematic reviews. The language of the search is limited to English and Chinese.

The combination of Medical Subject Headings and free-text words will be used as search strategy in our study search. The final search strategy of each database will be decided by multiple searches and modifications. Retrieval and filtering will be done by two authors (Zhipeng Hu and Maoyi Yang) independently. The search process is shown in Table 1.

**Table 1:**
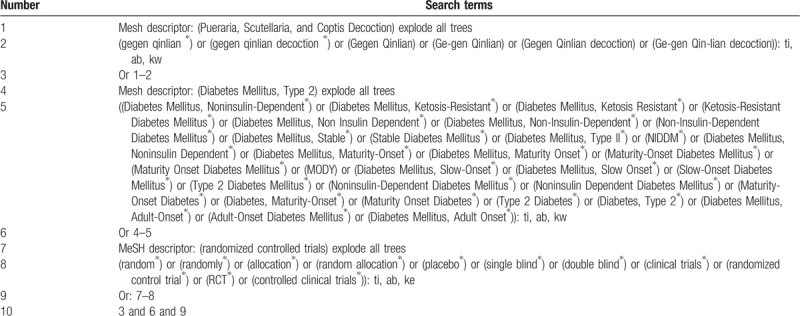
Example of Cochrane search strategy.

### 2.4 Study selection

**Figure 1:**
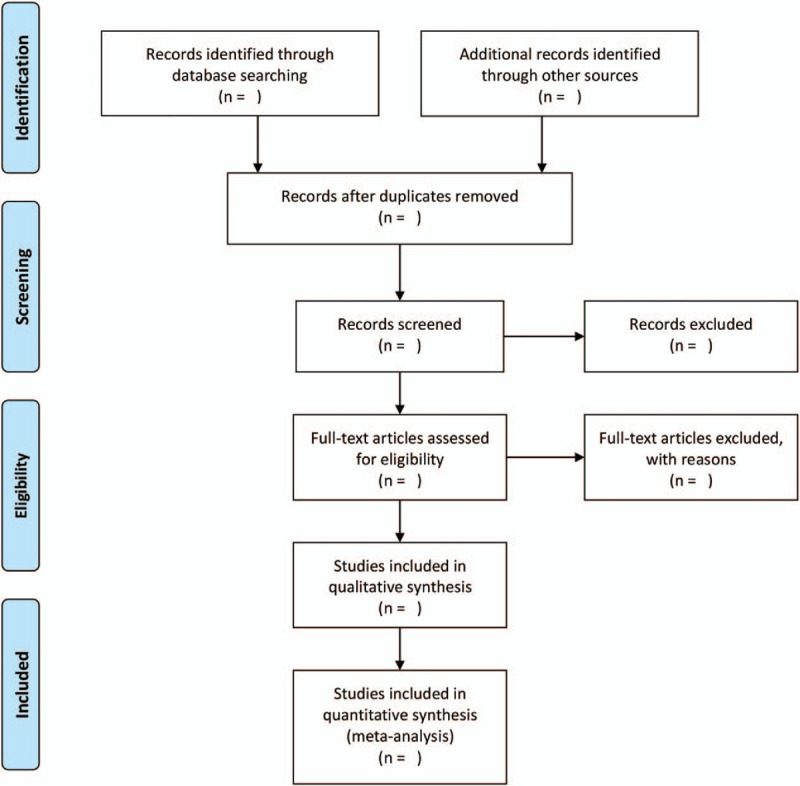
Flow chart of study selection.

We will use endnote X8 for Mac to manage retrieved articles. First, we remove duplicate studies by software. Then 2 authors (Zhipeng Hu and Rensong Yue) screen all the citations by reading the title and the abstract independently. Finally, the 2 authors will read the full text and determine the final qualified articles according to the inclusion and exclusion criteria. Disagreements in the process of study selection will be solved by a third author (Maoyi Yang). We will draw a flow chart showing all the processes of research selection (Fig. 1).

### 2.5 Data extraction

The data of qualified literature will be extracted into Microsoft Excel. We will extract the following information: title, name of the first author, the year of publication, the age and gender of patients, the course of disease, the intervention and the number of people in the experimental and control group, the treatment duration, outcomes and adverse effects. If there is a lack of necessary information in the article, we will email the corresponding author of the study for more detailed explanation. If the author doesn’t reply to the email or can’t provide the information we need, we will then exclude the study for a lack of key information.

### 2.6 Risk of bias assessment

According to the guidance of Cochrane Handbook for Systematic Reviews of Interventions, 2 authors (Maoyi Yang and Rensong Yue) will independently evaluate the methodological quality of the study from seven domains: random sequence generation, assignment concealment, participants and personnel blinding; outcome evaluation blinding; incomplete outcome data; selective reporting and other sources of bias. There are three classifications for each domain: “high risk” or “low risk” or “unclear risk”.^[13]^ discrepancies between the two authors will be solved by a third author (Zhipeng Hu).

### 2.7 Data analysis

We will use Review Manager Version 5.3 and stata 14.0 software for Mac to complete our meta-analysis. In this study, the effect size of binary variable will be expressed as risk ratio and 95% confidence interval and a mantel-haenszel method will be used to calculate them. The effect size of continuous variables will be used as mean difference or standardized mean difference and 95% confidence interval. Data heterogeneity test will be assessed by Cochrane *X*^2^ and *I*^*2*^ tests.^[14]^ If *P* < .05 and *I*^*2*^ > 50%, then the heterogeneity is substantial, and we will use the random effect model. If *P* > .05 and *I*^2^ < 50%, it is considered that the study included is homogeneous, and the difference between them can be ignored, and the fixed effect model will be adopted. If there is great heterogeneity in the included studies and quantitative synthesis is not appropriate, the results will be presented in the form of tables and charts.

### 2.8 Subgroup analysis

We build 3 hypotheses for subgroup analysis: Disease status at baseline, duration of intervention, type of concomitant medication.^[15]^ We will conduct our subgroup analysis according to preset subgroup hypotheses. If there are enough studies included, then meta- regression will be further conducted to explore the heterogeneity between studies.^[16]^

### 2.9 Sensitivity analysis

We will conduct sensitivity analysis on the results to evaluate the stability of the results. We will exclude the data contained in the analysis 1 by 1, and then summarize and analyze the data again. The difference between the recombined data and the original data is obtained. In this way, we can evaluate the impact of individual study on the overall results and whether the results we get are reliable.

### 2.10 Publication bias assessment

If the final number of original studies included is greater than 10, we will assess he publication bias of the study through funnel plot and egger test.^[17,18]^ If *P* < .05, it is considered that the included study has publication bias.

### 2.11 Summary of finding tables

Finally, the 2 researchers will evaluate the quality of evidence by Grading of Recommendations Assessment, Development and Evaluate system. The specific content will be carried out from 5 aspects: certainty assessment, number of patients, effect, certainty and importance. The quality of evidence can be classified as “high,” “medium,” “low” or “very low.”

### 2.12 Patient and public involvement

No patients and the public will be involved in the study.

### 2.13 Ethics and dissemination

Since our research is a meta-analysis of published data, ethical approval is not needed. Our findings will also be published in peer-reviewed journals.

## 3 Discussion

T2DM is 1 of the most common endocrine and metabolic diseases. Relative or absolute deficiency of human insulin secretion is the main cause of the disease.^[19]^ In recent years, with the continuous improvement of the quality of life, the number of diabetic patients is increasing, and their age become more youthful. This disease has become the third largest non-infectious disease after cardiovascular disease and tumor.^[1]^With the development of T2DM, many complications can occur in patients, such as nephropathy, retinopathy, cardio cerebrovascular disease and neuropathy, which will decrease the quality of life of patients and increase the mortality.^[20–22]^ The increasing number of people with diabetes has brought a heavy burden to the global health system. In China, the number of people with diabetes has been the rank first in the world for a long time.^[23]^ How to make better use of Chinese herbal medicine to play a therapeutic role in diabetes is the focus of our research. PSCD is a classic formula of traditional Chinese medicine, which has a long history in the treatment of diabetes. Researchers found that the combination therapy of PSCD and metformin is better than metformin alone in the treatment of T2DM.^[24]^ PSCD has the effect of reducing blood glucose, lipid and weight. In addition, PSCD can reduce the adverse effects caused by metformin.^[25]^ However, these studies have some shortcomings, such as small sample size, defective research design and inconsistencies in treatment course. Meanwhile, there is no systematic and comprehensive summary of the existing clinical evidence, which limits the clinical application of PSCD. In this study, we will conduct a systematic review and meta-analysis to provide high-quality evidence-based medical support for the clinical use of PSCD.

In this study, all of us will work reasonably and adopt scientific methods to draw more accurate and reliable conclusions. First, we will conduct subgroup analysis and regression analysis to explore the possible heterogeneity between the studies. In order to avoid meaningless post-hoc analysis, we will conduct subgroup analysis according to preset subgroup hypotheses. Then we will evaluate the subgroup analysis according to the reliability criteria of the subgroup analysis. Finally, we will classify the existing evidence by Grading of Recommendations Assessment, Development and Evaluate system, provide better guidance for clinical use and basis for further study of PSCD.

## Author contributions

**Conceptualization:** Maoyi Yang, Rensong Yue.

**Data curation:** Maoyi Yang, Zhipeng Hu

**Formal analysis:** Maoyi Yang, Zhipeng Hu

**Investigation:** Maoyi Yang, Rensong Yuen.

**Methodology:** Maoyi Yang, Zhipeng Hu.

**Project administration:** Rensong Yue

**Software:** Zhipeng Hu.

**Visualization**: Maoyi Yang, Zhipeng Hu.

**Writing – original draft:** Maoyi Yang.

**Writing – review and editing:** Rensong Yue.

Rensong Yue orcid: 0000-0002-4417-3312.
